# Oncolytic Adenovirus Coding for a Variant Interleukin 2 (vIL-2) Cytokine Re-Programs the Tumor Microenvironment and Confers Enhanced Tumor Control

**DOI:** 10.3389/fimmu.2021.674400

**Published:** 2021-05-18

**Authors:** Dafne C. A. Quixabeira, Sadia Zafar, Joao M. Santos, Victor Cervera-Carrascon, Riikka Havunen, Tatiana V. Kudling, Saru Basnet, Marjukka Anttila, Anna Kanerva, Akseli Hemminki

**Affiliations:** ^1^ Cancer Gene Therapy Group, Translational Immunology Research Program, University of Helsinki, Helsinki, Finland; ^2^ TILT Biotherapeutics, Helsinki, Finland; ^3^ Pathology, Finnish Food Authority, Helsinki, Finland; ^4^ Department of Obstetrics and Gynecology, Helsinki University Central Hospital, Helsinki, Finland; ^5^ Helsinki University Hospital Comprehensive Cancer Center, Helsinki, Finland

**Keywords:** oncolytic virus, IL-2 variant, anti-tumor immunity, tumor microenvironment (TME), virotherapy in cancer

## Abstract

The notion of developing variants of the classic interleukin 2 (IL-2) cytokine has emerged from the limitations observed with the systemic use of human IL-2 in the clinic: severe adverse events accompanied by low therapeutic response rate in treated patients. Modifications made in the IL-2 receptor-binding structure leads to preferential binding of IL-2 variant cytokine to receptors on effector anti-tumor lymphocytes over T regulatory (TReg) cells. Because of their inherent immunogenicity, oncolytic adenoviruses are useful for expression of immunomodulatory molecules in tumors, for induction of a pro-inflammatory state in the tumor microenvironment. In the present study, we constructed an adenovirus coding for an IL-2 variant (vIL-2) protein, Ad5/3-E2F-d24-vIL2. Functionality of the new virus was tested *in vitro*, and anti-tumor efficacy and mechanism of action studies were performed in immunocompetent hamsters bearing pancreatic tumors. Ad5/3-E2F-d24-vIL2 treatment elicited efficient anti-tumor response, with 62.5% monotherapy complete response. Moreover, it promoted substantial repression of genes associated with myeloid cells mediated immunosuppression (*CD11b, ARG1, CD206*). This was seen in conjunction with upregulation of genes associated with tumor-infiltrating lymphocyte (TIL) cytotoxicity (*CD3G, SAP, PRF1, GZMM* and *GZMK)*. In summary, Ad5/3-E2F-d24-vIL2 demonstrates therapeutic potential by counteracting immunosuppression and in efficiently coordinating lymphocytes mediated anti-tumor response in immunosuppressive tumors. Thus, Ad5/3-E2F-d24-vIL2 is a promising candidate for translation into clinical trials in human immunosuppressive solid tumors.

## Introduction

Recombinant human interleukin 2 (IL-2), also known as aldesleukin, was the first immunomodulatory protein approved for cancer treatment (1). IL-2 therapy introduced a new perspective on cancer treatment: instead of affecting cancer cells directly, it promotes anti-tumor responses through the stimulation of the host immune system (2, 3). The target was now normal cells instead of malignant cells. Effector CD8+ cytotoxic and CD4+ helper lymphocytes exert anti-tumor effects upon T-cell receptor (TCR) recognition of antigens and proliferative stimulation by IL-2 protein (4). However, retrospective clinical data on metastatic renal cell carcinoma patients who received high-dose IL-2 therapy showed an overall objective response rate of only 20%, including complete and partial responders (5). Additionally, high dose IL-2 therapy is associated with severe toxicity, which has limited its broader use in the clinic (6).

From the immunotherapeutic perspective, the main caveats associated with the use of systemic recombinant IL-2 therapy relate to low therapeutic levels in the tumor microenvironment (TME) and the activation of immunosuppressive cells, such as TRegs (3, 7). These observations can be explained by the short half-life of IL-2 in human serum, and its consumption by TRegs (3, 7). Also, toxicity from normal tissues can limit dosing before sufficient concentrations can be reached in the tumor (3, 4). While helper and effector T cells have immune-effector mechanisms, TRegs exert a pivotal role in limiting immune responses and promoting self-tolerance (8). In cancer immunotherapy, however, TRegs can expand considerably, leading to undesirable immunosuppression at the tumor (7, 9). Stimulation of TRegs is explained by the abundance of high-affinity IL-2 receptor on those cells, constituted by the subunits α (CD25), β (CD122), and γ (CD132) (10). In contrast, CD8+ cytotoxic and CD4+ T helper cells only transiently express high-affinity IL-2Rαβγ upon activation. TRegs are one of the few cell types that express IL-2Rαβγ constitutively, and whose functionality relies on a trimeric IL-2 receptor.

These learnings have motivated the development of IL-2 variant (vIL-2) proteins for overcoming these limitations, while preserving the immunotherapeutic benefits of wild type (wt) IL-2 (9). In some embodiments, modifications made in the IL-2 gene result in increased binding affinity to subunit β (CD122) of IL-2R, removing the functional need for variant IL-2 to bind to the CD25 subunit present in the IL-2 receptor (IL-2R) (9). If CD25 binding is not required, only cells not dependent on IL-2Rα binding will proliferate (9). These cells include CD8+ cytotoxic and CD4+ helper T cells (11–13). Some studies have shown promising pre-clinical data with improvement in tumor regression, and in the ratio of effector to TReg cells (9, 13). Some vIL-2 approaches have additionally reported Phase I and II data, with good safety, extended half-life in patient serum, and hints of objective response (11, 14).

Previously, we have demonstrated the advantages of using armed oncolytic adenoviruses to deliver therapeutic levels of different cytokines into tumor lesions, over traditional systemic cytokine administration (15, 16). In fact, in pre-clinical and clinical studies, adenoviruses armed with cytokines such as GMCSF, TNFa and IL-2 conferred improved anti-tumor response, with enhanced effector CD8+ T and helper CD4+ T cell trafficking into tumors, when compared to their unarmed versions (15–18). Moreover, genetically modified oncolytic adenoviruses promote selective infection and lysis of cancer cells, resulting in activation of damage and pathogen associated pattern recognition receptors in the microenvironment (15, 19). This helps convert immunosuppressive tumors to a pro-inflammatory state, conducive to anti-tumor response (17). Further, the host anti-viral response amplifies the overall anti-tumor response (20).

In the present study, we engineered an adenovirus coding for a human vIL-2 protein (Ad5/3-E2F-d24-vIL2) to promote anti-tumor response through the selective stimulation of effector T cells in the context of immunosuppressive solid tumors. We demonstrated Ad5/3-E2F-d24-vIL2 virus is functional in lysing of and in expressing the vIL-2 transgene in infected cancer cells. Mechanistically, treatment response was associated with reduced expression of genes linked to suppressive myeloid phenotypes (*CD11b, ARG1, CD206*) in the tumor microenvironment. Concurrently, we observed increased expression of genes associated with tumor infiltrating lymphocyte (TIL) cytotoxicity (*CD3G, SAP, PRF1, GZMM and GZMK*).

## Materials and Methods

### Cell Lines

Human cancer cell lines A549 (lung cancer) and OVACR3 (ovarian cancer) were purchased from American Type Culture Collection (ATCC) (Manassas, USA). The Syrian hamster cancer cell line DDT1-MF2 (leiomyosarcoma) was a kind gift from Dr. William Wold. The Syrian hamster HapT1 (PDAC cancer cell line) was obtained from Leibniz Institute (DSMZ, Braunschweig, Germany). All cell lines were cultured under recommended conditions, and cultures were passaged three to four times prior to use in the experiments.

### Virus Construct

All viruses included in the present study utilized the Ad5/3-E2F-d24 backbone structure (21). The virus construct coding for human IL-2 protein, Ad5/3-E2F-d24-hIL2, has been previously described (17). For the generation of the oncolytic adenovirus armed with a human vIL-2, Ad5/3-E2F-d24-vIL2, we inserted five point mutations in the IL-2 sequence at positions L80F, R81D, L85V, I86V and 92 I92F. This modified gene was then inserted under the E3 gene promoter region, replacing gp19k and 6.7k as described before (17).

### Virus Functionality

Ad5/3-E2F-d24-vIL2 cytotoxic capability was evaluated *in vitro* through a cell viability assessment. Human A549 and OVCAR3, and hamster DDT1-MF2 cell lines were platted in triplicates, infected with a crescent concentration of Ad5/3-E2F-d24, Ad5/3-E2F-d24-hIL2, or Ad5/3-E2F-d24-vIL2 viruses, or left uninfected (mock group). Cell viability was measured after 3 (A549) or 5 days (OVCAR3 and DDT1-MF2) of incubation with MTS assay by adding 20% CellTiter 96 Aqueous One Solution (Promega, Madison, WI, USA) on the cells, and reading the final absorbance at 490nm after two hours of incubation. Supernatants from virus infected cells were collected, and vIL-2 transgene levels were detected through human IL-2 flex set (BD Biosciences, CA, USA), following manufacturer´s instructions. Samples were measured with Accuri C6 flow cytometer (BD Biosciences, La Jolla, CA, USA), and analyzed with FCAP Array software (BD Biosciences, La Jolla, CA, USA).

### Animal Experiments

Immunocompetent male Syrian golden hamsters, 5 weeks old, (Envigo, Indiana, USA) were used as a pre-clinical model for the *in vivo* studies. For the mechanism of action and survival experiments, hamsters were engrafted on their left lower back with a single injection of 2x10^6^ HapT1 cells. Tumor growth was followed until day 5, when 5 to 6 mm diameter was reached. Animals were randomized into one of the treatment groups: Ad5/3-E2F-d24, Ad5/3-E2F-d24-hIL2, and Ad5/3-E2F-d24-vIL2. A mock group was included as an experimental control. Virus-treated groups received 1x10^9^ vp intratumoral injections, while the mock group only PBS was administered. A digital caliper was used to measure the tumor progression across the experimental days. Tumor volumes were calculated as (length x width^2^)/2. The tumor volume in percentage was obtained through the normalization of the daily tumor volumes to their respective day 0 tumor volume.

For the mechanism of action studies (n=5 per group), hamsters received four rounds of virus treatment before they were euthanized on day 16. Tumors and selected organs were collected for further analysis. Concurrently, a survival experiment (n=8 per group) was carried out in parallel following the same conditions as aforementioned. In this embodiment, animals were treated with eleven additional rounds of virus injections every five days, while the tumors were visible, and were observed for tumor progression up to 160 days, when the experiment was ended. Animals were euthanized whenever the maximum allowed tumor volume (22.0mm) was reached in the longest tumor diameter, tumors developed ulcers, or whenever the animals´ wellbeing was compromised.

Complete responders from the survival experiment were further assessed for specific anti-tumor responses. Cured animals were re-challenged with of 2x10^6^ HapT1 cells, and challenged with a new cancer cell line, 2.5x10^5^ DDT1-MF2 cells, engrafted on their upper left and right flanks, respectively. As a control group, naïve hamsters (n=3) were used. Tumor development was observed for two weeks.

### Flow Cytometry

Hamster tumor samples collected on day 16, were mechanically disrupted into single cell suspensions with a tissue grinder and processed for flow cytometry analysis. Samples were stained with antibodies for CD8+ (PE, 12-0080-82; eBioscience, San Diego, CA, USA), CD4+ (PE-Cyanine 7, 25-0041-82; eBioscience, San Diego, CA, USA), and MHC II+ cells (FITC, 11-5980-82; eBioscience, San Diego, CA, USA). NK+ cells were labelled with the polyclonal antibody anti-Asialo-GM1 (Alexa Fluor-488, 53-6507-80; eBioscience, San Diego, CA, USA), and macrophages and dendritic cells (Mac-2) cells with anti-Galectin 3 (PE, 12-5301-82; eBioscience, San Diego, CA, USA) as described before (17). Cell fluorescence was detected using Sony SH800Z cytometer (Sony, Tokyo, Japan) upon the acquisition of the 100,000 events per sample. Cell data processing and gating were performed with FlowJo v.10.6.1 (Ashland, OR, USA).

### Histopathology Analysis

Selected tissues (livers, lungs, hearts, spleens, and kidneys) and tumors collected for histopathological analysis were fixed in 10% formalin, and routinely processed and paraffin embedded. Samples paraffin-blocks were sectioned into 5μm thickness slides and further stained with hematoxylin and eosin. A veterinarian pathologist examined the samples slides in a blind manner.

### Reverse transcriptase (RT)-Quantitative Polymerase Chain Reaction (qPCR)

Fragments of animal tumor samples harvested on day 16, were preserved in RNAlater (R0901; Sigma-Aldrich, St. Louis, USA), and stored in -20°C until further processing. Approximatelly, 15 to 20 mg of RNAlater stabilized tumor fragment from each animal was then purified with RNAeasy Mini Kit (74104; QIAGEN, Hilden, Germany) following the manufacturer´s instructions. The purified RNAs were used to synthetize cDNA using Quantitect Reverse Transcription Kit (205313; QIAGEN, Hilden, Germany) to be used for the relative quantification of the viruses’ transgene expression as well as for hamster IL-2 relative expression. The primers and probes used for the hamster endogenous IL-2 and the virus transgenes expression are described in the **Supplementary Table 1** (16, 22). Cycling conditions for the qPCR runs were used as previously established (22).

### Gene-Expression Analysis

RNA purified from Day 16 tumors were quantified with the Thermo Scientific NanoDrop™ 1000 Spectrophotometer (Thermo Fisher Scientific, Massachusetts, USA), and the RNA concentration of the samples were adjusted to 20ng/µl. NanoString nCounter^®^ gene expression analysis was performed on the RNA samples from all hamster tumors individually utilizing the nCounter^®^ Digital Analyzer (NanoString Technologies, Seattle, USA). Gene expression was assessed with a custom-panel designed for hamster cells containing 99 genes analysed by nSolver software 4.0 (NanoString Technologies, Seattle, USA). Differential expression is displayed as the values for each genes´ gene’s -log10 (p-value) and log2 fold change in the volcano plots. Likewise, differential expression as RNA counts (Log2) are displayed in the bar graphs. The expression level of each gene in the treatment groups was normalized to their corresponding genes in the control (mock) group.

### Statistical Analysis

Mixed-model analysis was performed to evaluate the tumor progression, using the transformed logarithmic normalized tumor volumes in SPSS v.25 (IBM, Chicago, IL, USA). GraphPad Prism v.8.4.2, (San Diego, CA, USA) was used for log rank Mantel-Cox on Kaplan-Meier survival curve, Pearson correlation, linear regression, unpaired t-test with Welch´s correction, and for the graphical representation of the data. The results were considered statistically significant when p<0.05.

### Ethical Statement

The animal experiments and procedures here described were performed in accordance with the Experimental Animal Experimental Board (ELLA) of the University of Helsinki and the Provincial Government of Southern Finland.

## Results

### An Oncolytic Adenovirus Expressing Human IL-2 Variant Protein Exerts Cytotoxic Effects in Cancer Cells

**Figure 1:**
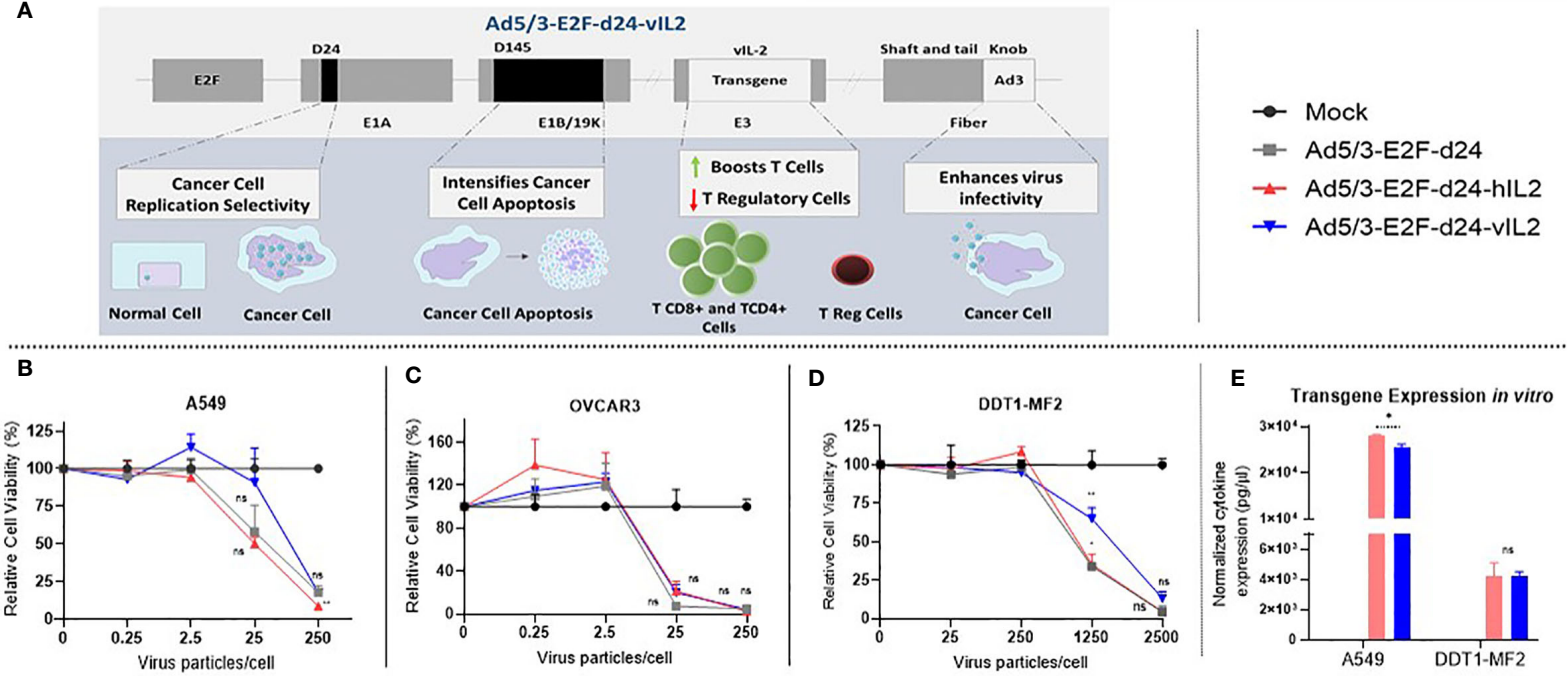
View on the Ad5/3-E2F-d24-vIL2 virus construct and functionality testing *in vitro*. **(A)** Graphical illustration of the features of the virus genome. **(B–D)** Relative cancer cell viability upon Ad5/3-E2F-d24-vIL2 virus infection for 3 (A549) or 5 days (OVCAR3 and DDT1-MF2). **(B)** Human lung cancer cell line (A549). **(C)** Human ovarian cancer cell line (OVCAR3). **(D)** Hamster leiomyosarcoma cancer cell line (DDT1-MF2). **(E)** Normalized transgene production in supernatants of virus-infected A549 and DDT1-MF2 cancer cells at 250 and 2500 vp, respectively. All data is presented as mean + SEM. *p < 0.05 and **p < 0.01. ns, not statistically significant.

Ad5/3-E2F-d24-vIL2 uses a backbone from adenovirus serotype 5 that carries the knob from serotype 3 (21). This modification improves transduction of cancer cells through desmoglein 2, which is highly expressed by most malignant cells (23). Additionally, a deletion in the E1B/19k region of the backbone was engineered to intensify apoptosis induction and TNFa mediated cell death in cancer cells. To restrict adenovirus replication to cancer cells, a 24-bp deletion (d24) was engineered in the constant region 2 of the E1A gene. Tumor specificity was further enhanced by the insertion of a tumor-specific E2F promoter upstream of the E1A region. Lastly, the vIL-2 gene was inserted in the partially deleted E3 gene region, resulting in a construct where vIL-2 expression is linked to virus replication **(** **Figure 1A** **)**. Since the replication of viruses of this type is strictly restricted to tumor cells, also transgene production is limited to tumors (17).

vIL-2 virus showed similar oncolytic capability in human and hamster cancer cells when compared to unarmed virus **(** **Figures 1B–D** **)**. Of importance, these results demonstrate that the virus intrinsic lytic capability was not compromised by the insertion of the vIL-2 transgene. Transgene production by the vIL-2 and wt IL-2 viruses was comparable in both human and hamster cancer cells. Nevertheless, in A549 cells wt IL-2 virus resulted in slightly higher IL-2 protein levels than vIL-2 virus (p=0.013) **(** **Figure 1E** **)**. Overall, Ad5/3-E2F-d24-vIL2 was validated as an expression vector for vIL-2 protein.

### IL-2 Variant Armed Oncolytic Adenovirus Treatment Promotes Tumor Control and Overall Survival *In Vivo*

**Figure 2:**
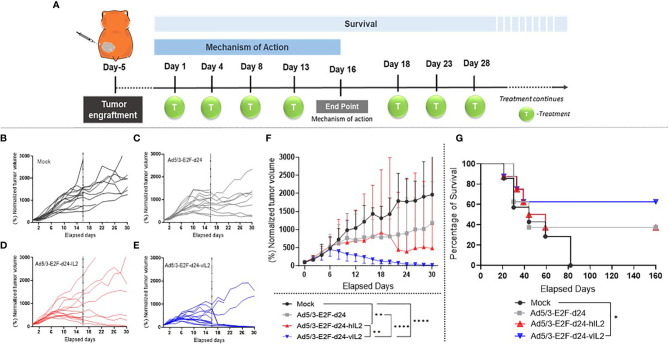
Anti-tumor effects mediated by viral treatments in a hamster pre-clinical model. **(A)** Illustration of the experimental design. **(B–E)** Normalized individual tumor volumes from mechanism of action studies were plotted until the end of the experiment (dashed line on day 16), while the animals enrolled in the survival experiment continued being measured up to day 30. **(F)** Graphical representation of median tumor size, with bars showing range, until day 30. The tumor volume in percentage was obtained through the normalization of the daily tumor volumes to their respective day 0 tumor volume. **(G)** Survival curve of all experimental groups until day 160. *p < 0.05, **p < 0.01, and ****p < 0.0001.

Local treatment injections were administered following the therapeutic scheme described in **Figure 2A**. Tumors treated with wt IL-2 virus showed significantly higher anti-tumor efficacy when compared with mock group (p=0.003). vIL-2 gave the best anti-tumor response compared to mock (p<0.0001), virus backbone (p<0.0001), and wt IL-2 virus (p=0.004) **(** **Figures 2B–E** **)**. As a complementary view to individual tumor size, median tumor size is shown in **Figure 2F**. Overall, these results demonstrate the anti-tumor potential of Ad5/3-E2F-d24-vIL2.

In survival analysis, the mock group had shortest survival, as all animals were dead by day 82. Unarmed virus and wt IL-2 virus groups had the same proportion of survivors, with 3 out of 8 animals alive at the end of the experiment (37.5%). However, 2 out of 3 animals treated with the unarmed virus still had visible tumors at the end of the experiment, while all 3 animals in the wt IL-2 group were tumor free. Ad5/3-E2F-d24-vIL2 was the only treatment able to statistically significantly prolong survival when compared to mock (p= 0.044) **(** **Figure 2G** **)**. 62.5% of animals were alive at the end of the experiment.

### Treatment With IL-2 Variant Virus Promotes Moderate T Cell Infiltration And High MHC-II Expression in the Tumor Microenvironment

**Figure 3:**
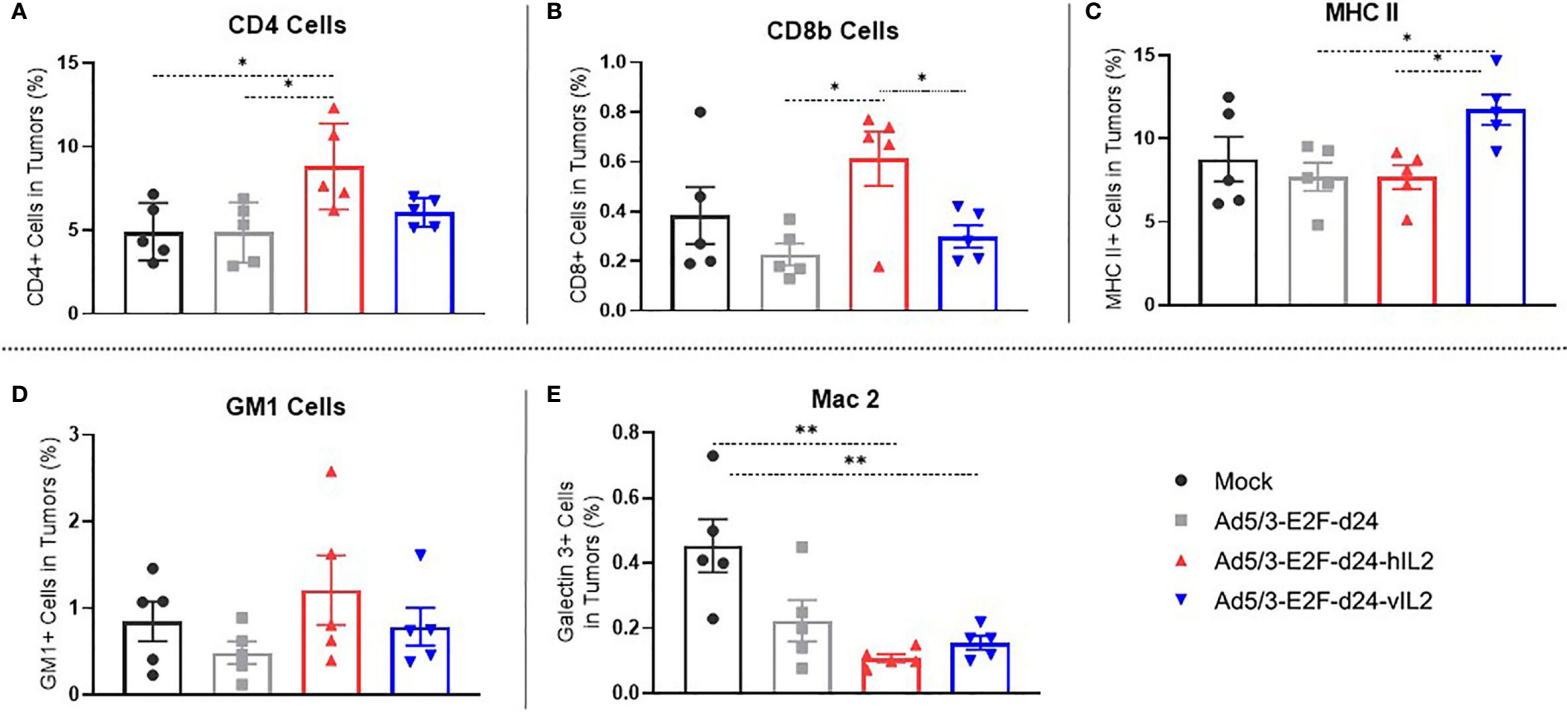
Phenotype study of the immune cells constituting the microenvironment of treated tumors, by flow cytometry. The graphs show the percentage of **(A)** tumor infiltrating CD4+ T cells, **(B)** CD8+ T cells, **(C)** MHC II expressing cells, **(D)** natural killer cells, and **(E)** macrophages out of singlets. Data is presented as mean + SEM. *p < 0.05, and **p < 0.01.

CD4+ T cells were more frequent in wt IL-2 virus treated tumors than in mock (p=0.025), virus backbone (p=0.025), and vIL-2 virus groups (not significant) **(** **Figure 3A** **)**. Interestingly, the highest frequency of CD8+ T cells was also encountered in the wt IL-2 virus group, when compared to unarmed virus (p=0.021) and vIL-2 (p=0.043) groups **(** **Figure 3B** **)**. Unfortunately, the lack of hamster-specific or cross-reactive antibodies prevented more precise characterization of these T cell populations.

vIL-2 virus treated tumors presented a statistically significant higher frequency of MHC II expressing cells in comparison to unarmed virus (p=0.012) and wt IL-2 virus (p=0.009) **(** **Figure 3C** **)**. The results for macrophages (Mac 2) and natural killer (GM-1+) cells were similar in virus treated groups **(** **Figures 3D, E** **)**. Overall, these results show that anti-tumor efficacy seen in vIL-2 treatment is associated with a modest frequency of CD8+ and CD4+ T cells and with a high expression of MHC-II positive infiltrating cells in the tumor microenvironment.

### Moderate Level Of Necrosis Is Associated With Tumor Regression and Increased Cytokine Production in Ad5/3-E2F-d24-vIL2 Treated Tumors

**Figure 4:**
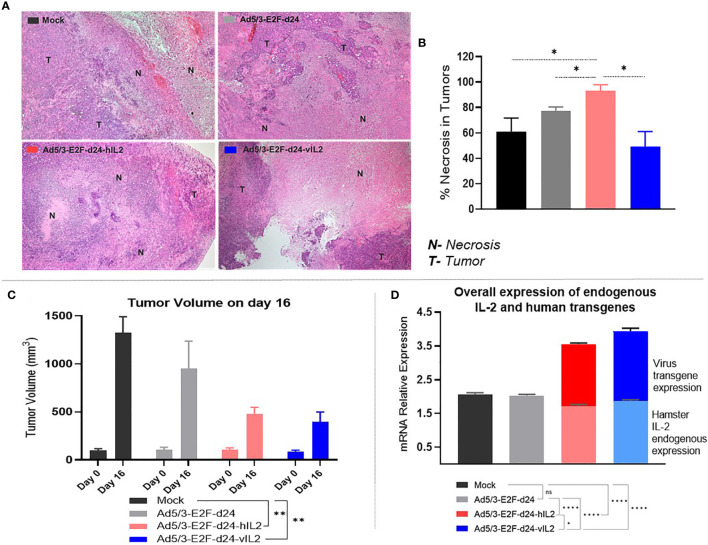
View on tumor necrosis levels, anti-tumor response, and cytokine production in treated tumors. **(A)** Representative pictures of tumor tissues slides indicating necrotic (N) and tumor (T) areas in the experimental groups. **(B)** Percentage of necrosis levels identified across groups. **(C)** Tumor volume (mm^3^) comparison on days 0 and 16. **(D)** Endogenous hamster wt IL-2 (bottom graphs) and virus delivered transgenes (upper graphs) relative mRNA expression. All data is presented as mean + SEM. *p < 0.05, **p < 0.01, and ****p < 0.0001. ns, not statistically significant.

The highest necrosis score was observed in the wt IL-2 virus group (**Figure 4A**), 93% of the tumor tissue (**Figure 4B**). This was statistically significantly higher when compared to mock (p=0.037), virus backbone (p=0.031), and vIL-2 virus (p=0.018) groups. On average, Ad5/3-E2F-d24-vIL2 resulted in 49% necrosis in treated tumors **(** **Figure 4B** **)**. Nevertheless, the lower necrosis levels did not compromise its ability to promote efficient tumor regression, compared to the other virus treatment groups and mock (p=0.003), on day 16 **(** **Figure 4C** **)**. Interestingly, these results demonstrate that Ad5/3-E2F-d24-vIL2 treatment efficiently stimulates anti-tumor effects with less prominent necrosis, in comparison to armed wt IL-2 virus. None of the treatments caused histopathological changes in normal hamster tissues (lungs, hearts, kidneys, livers, and spleens).

Tumor samples were assessed at the mRNA level for the expression of hamster endogenous IL-2 production and transgene expression **(** **Supplementary Figures 1A, B** **)**. To obtain an overview on the global cytokine production, endogenous hamster IL-2 plus virus transgenes relative expression levels were then combined. Interestingly, vIL-2 virus was capable of stimulating the highest global relative expression of hamster IL-2 plus vIL-2 cytokines in the tumor microenvironment: mock (p<0.0001), virus backbone (p<0.0001), and wt IL-2 virus (p=0.026) **(** **Figure 4D** **)**.

### Ad5/3-E2F-d24-vIL2 Treatment Stimulates Up Regulation of Genes Associated With Anti-Tumor Efficacy

**Figure 5:**
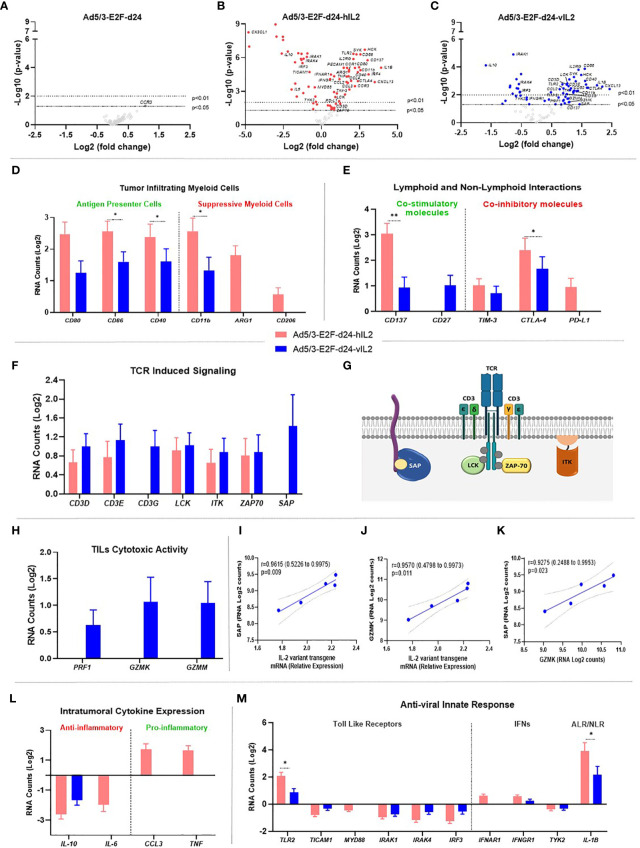
Molecular gene expression analysis in the virus treated tumors. **(A–C)** Volcano plot graphs showing each gene’s -log10(p-value) and log2 fold change with expression change upon **(A)** Ad5/3-E2F-d24, **(B)** Ad5/3-E2F-d24-hIL2, and **(C)** Ad5/3-E2F-d24-vIL2 treatments. Statistically significant genes fall at the top of the plot above the horizontal lines, and differentially expressed genes fall to left and right sides on the X axis. The dashed horizontal lines represent the significance levels thresholds: p<0.05 or p<0.01. **(D)** Log2 mRNA expression of genes associated to antigen presenting cells (left side) and to suppressive myeloid cells (right side). **(E)** Lymphoid and non-lymphoid interactions: mRNA (Log2) expression study on co-stimulatory (left side) and co-inhibitory molecules (right side). **(F)** Log2 mRNA expression of genes associated to TCR induced signaling. **(G)** Graphical illustration of TCR signaling involved proteins on the surface of lymphocyte cells. **(H)** Log2 mRNA expression of TIL cytotoxicity genes. **(I)** Correlation between SAP and vIL-2 transgene expression levels. **(J)** Correlation between GZMK and vIL-2 transgene levels. **(K)** Correlation between SAP and GZMK expression levels. **(L)** Intratumoral expression of anti-inflammatory cytokines (left side) and pro-inflammatory cytokines (right side) as mRNA (Log2) levels. **(M)** Anti-viral innate immune response related to TLRs (left side), IFNs (middle), and inflammasome signaling *via* AIM2-like receptors (ALRs) and NOD-like receptors (NLRs) (right side) as mRNA (Log2) expression. All data is presented as mean + SEM. *p < 0.05, **p < 0.01.

In order to get further insights into the mechanisms of Ad5/3-E2F-d24-vIL2 treatment, transcriptomic analyses were performed on tumor samples from day 16 (**Supplementary Table 2**). Cytokine-armed viruses produced marked changes in gene expression levels, compared to the unarmed backbone which up-regulated only a single gene (*CCR3*). In the wt IL-2 virus group, a total of 73 genes showed significant changes in their expression levels. Similarly, vIL-2 virus significantly changed 62 genes **(** **Figures 5A–C** **)**. Also, the list of genes impacted was quite different between wt and variant IL-2, underlining biological differences resulting from engineering of the transgene.

### IL-2 Variant Armed Adenovirus Treatment Strengthens Antigen Presenting Cells and Weakens Immunosuppressive Myeloid Cells Effects in the Tumor Microenvironment

Wild type IL-2 virus treatment induced significantly higher expression of antigen presenting cell related genes (*CD80*, *CD40*; p=0.025, *CD86*; p=0.027) than vIL-2 virus. Interestingly, wt IL-2 virus enhancing effects were even more prominent on the immunosuppressive compartment. Expression levels of myeloid derived suppressor genes (*CD11B*; p=0.040, *CD206*, *ARG1*) were significantly upregulated with wt IL-2 virus treatments. Of note, in vIL-2 treated tumors only moderate expression of *CD11B* was detected, while *CD206* and *ARG1* remained unaltered **(** **Figure 5D** **)**. In the wt IL-2 group, increased expression was observed in genes associated with migration and degranulation of granulocytes (*PECAM1, HCK*; p=0.002, **Supplementary Figure 2** **)**. Together, these results imply that the vIL-2 virus is able to break down immunosuppressive myeloid forces, and selectively stimulates antigen presenting cells at the tumor site.

### Modest Co-Stimulatory Signals Associate With Improved Tumor Regression in IL-2 Variant Treated Tumors

vIL-2 adenovirus modestly increased gene levels of known co-stimulatory factors (*CD137, CD27*) in treated tumors. On the other hand, wt IL-2 virus expressed significant higher levels of *CD137* (p=0.007), while *CD27* did not change after treatment. Correspondingly, the results from co-inhibitory molecules show that wt IL-2 virus treated tumors had higher levels of *TIM-3, CTLA-4* (p=0.043), and *PD-L1* genes compared to vIL-2. Of note, *PD-L1* (*CD274*) gene expression remained unchanged in vIL2 virus treatment group, while it did increase with the wt IL-2 virus **(** **Figure 5E** **)**. In summary, our data indicate that in vIL-2 virus-treated tumors, lymphoid and non-lymphoid cell interactions were associated with improved tumor rejection.

### Ad5/3-E2F-d24-vIL2 Virus Boosts TIL Toxicity Through Effective T Cell Receptor (TCR) Anchoring

All studied genes associated with expression of TCR signaling molecules were upregulated following vIL-2 virus therapy. Specifically, CD3 co-stimulatory complex genes (*CD3D, CD3E, CD3G*) were found at higher levels following vIL-2 virus than wt IL-2 virus. Of note, *CD3G*, which is associated with expression of the TCR complex on the cell surface, was significantly upregulated in vIL-2 group, whereas it remained unaltered in the wt IL-2 group. Additionally, molecules involved in TCR-MHC transduction of immunological signals (*LCK, ITK, ZAP70*) were also upregulated by vIL-2, although no statistical significance was found. Surprisingly, an important molecule responsible for the docking of the TCR with MHC expressing antigen (SAP) was exclusively upregulated in the vIL-2 virus group **(** **Figures 5F, G** **)**. This data set suggests that Ad5/3-E2F-d24-vIL2 supports stable immunological synapsis in T effector cells at the tumor site.

Likewise, striking results were identified in the cytotoxicity profile of TILs. Genes associated with cytotoxic lymphocyte degranulation (*PFR1, GZMK, GZMM*) were increased only with Ad5/3-E2F-d24-vIL2 **(** **Figure 5H** **)**.

Therefore, further evaluation of the effect of vIL-2 on TCR-MHC anchoring protein gene (*SAP*) and a lymphocyte effector lysing activity gene (*GZMK*) was performed. Interestingly, a strong positive correlation was observed between *vIL-2* and *SAP* (p=0.009; r=0.9615 [0.5226 to 0.9975]) and *GRZMK* (p=0.011; r=0.9570 [0.4798 to 0.9973]) **(** **Figures 5I, J** **)**. Likewise, similar positive correlation was observed between *SAP* and *GZMM* expression (p=0.009; r=0.9615 [0.5226 to 0.9975]) **(** **Figure 5K** **)**. Taken together, these results suggest that the immunological molecular mechanisms elicited by the vIL-2 virus, to enhance anti-tumor responses, is mediated by TILs.

### Armed Adenoviruses Stimulate Pro-Inflammatory Cytokine Expression in Treated Tumors

Expression of anti-inflammatory cytokine genes (*IL-10, IL-6*) was significantly downregulated with both armed viruses. Cytokines with pro-inflammatory activity (*CCL3, TNF*), however, were upregulated only in the wt IL-2 group **(** **Figure 5L** **)**. Ad5/3-E2F-d24-vIL2 treatment did not induce noteworthy cytokine or chemokine changes **(** **Supplementary Figure 3** **)**.

### Anti-Viral Immune Responses Mediated by Armed Viruses

Among toll like receptor (TLR), IFN signaling, and inflammasome pathways, only *TLR2* was up-regulated (both armed adenovirus groups). Other TLR signaling mediators (*TICAM1, MYD88, IRAK1, IRAK4, IRF3*) were downregulated. Of note, an important mediator for TLR adenovirus immunity (*TLR9*) did not meet the minimum expression level inclusion criteria. In the IFN response axis, a partial up-regulation of some IFN receptors (*IFNAR1, IFNGR1*) was observed while a critical pathway signaling mediator (*TYK2*) was downregulated in the armed adenovirus groups **(** **Figure 5M** **)**. In the inflammasome pathway, IL-1β expression was significantly higher with wt IL-2 virus than with the vIL-2 virus (p=0.030).

### Ad5/3-E2F-d24-vIL2 Treatment Promotes Anti-Tumor Immune Protection in Tumor Re-Challenge

**Figure 6:**
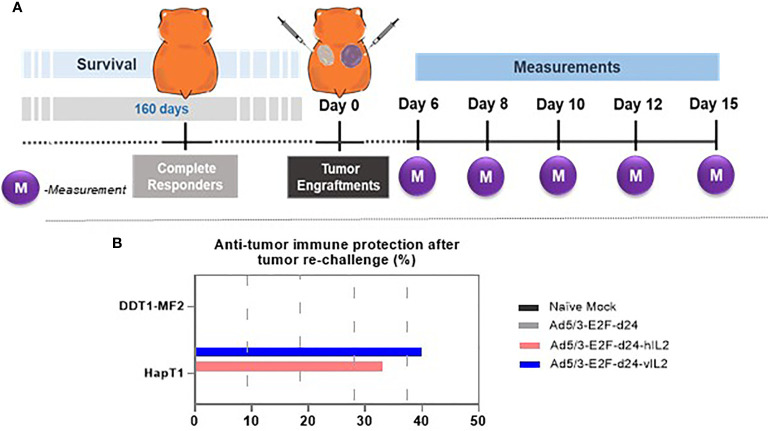
Anti-tumor immune protection experiment in complete responders. **(A)** Experimental design illustration of tumor engraftments and measurements. **(B)** Anti-tumor immune protection provided by the treatment groups after the tumor re-challenge.

Animals that survived until day 160 without visible tumors were assigned to tumor re-challenge **(** **Figure 6A** **).** Six days after the re-challenge, HapT1 tumors started to grow, although tumor regression was observed in the groups whose tumors had been cured with armed viruses **(** **Figure 6B** **)**. In contrast, all DDT1-MF2 tumors, for which animals were naïve to, progressed in all experimental groups showing no sign of tumor control. Of note, vIL-2 virus promoted anti-tumor memory protection in 40.0% of the re-challenged animals. Although not statistically significant, this result was superior to the one obtained with wt IL-2 virus (33.0%), and unarmed virus (0.0%) **(** **Figure 6B** **)**.

## Discussion

Engineered versions of the classic IL-2 cytokine have brought new excitement into the cytokine immunotherapy field. Nevertheless, beyond TRegs, the mechanisms of variant IL-2 cytokines have not been discussed much. Here, we present a strategy for mitigation of tumor immunosuppression beyond TReg, utilizing an oncolytic adenovirus to locally express a human vIL-2 cytokine.

We demonstrate that the vIL-2 transgene represents a therapeutic advantage in comparison to wild type IL-2 as an arming device. In fact, the immunosuppressive nature of the PDAC tumor model (24) used here did not hamper Ad5/3-E2F-d24-vIL2 virus treatment response. On the contrary, vIL-2 virus resulted in the best anti-tumor response, even over its wild type counterpart. Together, these results highlight the prospective therapeutic potential of the vIL-2 virus for the treatment of immunosuppressive tumors such as pancreatic cancer.

Importantly, the benefits observed with Ad5/3-E2F-d24-vIL2 therapy became more pronounced over time, with 62.5% of the treated animals presenting long term complete response. This result is particularly relevant considering that vIL-2 virus was used as a monotherapy. Other approaches, such as PEGylated IL-2 (NKTR-214) did not achieve similar success when mouse breast and colon cancer tumors were treated as monotherapy (25). In fact, NKTR-214 therapy only showed a similar rate of complete responders when combined with CTLA-4 checkpoint blockade (25). Here, the oncolytic adenovirus platform seems to contribute more than direct tumor cells lysis, as it results in high local expression of transgenes, and in amplification of the immunological signal through epitope spreading (19, 26).

Histopathological analysis of tumor sections demonstrated that vIL-2 virus anti-tumor response promotes a lower level of cell necrosis than the wt IL-2 virus. Nevertheless, moderate necrosis levels did not mean less anti-tumor effects for Ad5/3-E2F-d24-vIL2. On the contrary, Ad5/3-E2F-d24-vIL2 exhibited a clearer tendency towards complete tumor regression. This was later confirmed in a survival experiment. It has been reported that in certain situations tumor necrosis can be associated with bad prognosis and tumor progression (27). Necrosis can release cancer-promoting factors like MMP1, HMGB1, and ICAM1, which induce microenvironment changes resulting in tumor aggressiveness (28, 29). Our data suggest that Ad5/3-E2F-d24-vIL2 utilizes mechanisms more refined than necrosis in tumor debulking.

One of the advantages of using a vectored system is prolonged spatially restricted high production of the transgene in tumor lesions with minimum leakage to healthy tissues (16, 17). The virus constructs used here express their transgene only in those cells that allow virus replication (*i.e.* tumor cells). Ad5/3-E2F-d24-vIL2 treatment promoted the highest global levels of total IL-2 (endogenous hamster IL-2 plus transgene expression) in treated tumors. This result demonstrates the outstanding capacity of the adenovirus vector in sustaining high transgene levels. Endogenous expression of hamster IL-2 was slightly lower with the armed viruses, probably due to negative feedback (10). It is noteworthy that TIM-3 expression associates with the regulation of wt IL-2 production (30). Indeed, we found that TIM-3 expression was inversely proportional to hamster IL-2 expression in tumors. However, negative feedback in IL-2 production did not affect efficacy.

Here, we show that Ad5/3-E2F-d24-vIL2 is a promising candidate to overcome two main caveats that have restricted the clinical use of wt IL-2 cytokine therapy: low therapeutic levels in the tumor lesion, and severe systemic toxicity (7, 31). We obtained high IL-2 levels in tumors while no abnormalities were observed in healthy organs.

vIL-2 is intended to stimulate effector T cells over TReg at the tumor site (9, 13). Indeed, this feature is often observed across different approaches utilizing IL-2 variants: high ratios of CD8+ T cells to CD4+ TReg cells in treated tumors (13, 25). Concurring with these results, vIL-2 resulted in fewer CD4+ T cells than wt IL-2. Unfortunately, a precise distinction of the CD4+ TReg subset population was not possible due to the limited availability of hamster reagents (17).

On the other hand, CD8+ T cells were not as abundant in Ad5/3-E2F-d24-vIL2 treated tumors as with Ad5/3-E2F-d24-hIL2. Differences on cytokine receptor interactions could explain these findings; vIL-2 binds to IL-2Rβγ with intermediate affinity, while wt IL-2 interacts with higher affinity to the trimeric IL-2Rαβγ, leading to a faster response to wt IL-2 cytokine stimuli (32, 33). Additionally, Au-Yeung and colleagues reported that wt IL-2 cytokine reduces the antigen stimulus tolerance threshold for TCR signaling in CD8+ T cells (34), meaning that CD8+ T-cell expansion might be more pronounced with wt IL-2 (34). This is in line with our observations on intratumoral CD8+ T-cell percentage. Of note, our data suggests enhanced tumor antigen presentation through MHC II expression in the Ad5/3-E2F-d24-vIL2 treated group. In summary, these results highlight that Ad5/3-E2F-d24-vIL2 mediated anti-tumor activity is associated with high CD8+ T cell efficiency and improved MHC II antigen presentation.

Importantly, the myeloid-cell compartment exerts a crucial role in the initiation, modulation, and suppression of tumor immunity (35, 36). In the present study, we observed that genes associated with antigen presentation (*CD80, CD86, CD40*) were up-regulated in both armed-virus groups. Interestingly, these findings support our flow cytometry MHC-II results, and together suggest that Ad5/3-E2F-d24-vIL2 is effective in antigen presentation to CD4+ T helper cells at tumors. For the virus coding for wt IL-2 (Ad5/3-E2F-d24-hIL2), however, these pro-inflammatory responses were potentially counteracted by suppressive genes (*CD11B, ARG1, CD206, HCK*) *(* 36). Especially CD11b has been associated with recruitment of myeloid cells with an immunosuppressive phenotype (*CD206, ARG1*) in human pancreatic tumor analyses (36). As a consequence, TAMs and granulocytes release mediators such as arginase 1 (*ARG1*) and hematopoietic cell kinase (*HCK*) that impair T-cell responses and promote tumor invasion in lung and in colon cancers (37, 38). Together, these data highlight the ability of Ad5/3-E2F-d24-vIL2 to reshape the tumor environment from an immunosuppressive towards a pro-inflammatory one.

From the co-stimulatory perspective, CD137 was up-regulated by both armed viruses. Nevertheless, the expression of CD137 expression in tumors is quite controversial (39, 40). While CD137 it is often associated with activation of T effector cells (40), recent clinical data revealed that CD8+ TILs expressing CD137 indicated a highly exhausted cell profile in hepatocellular carcinoma patients (41). In PDAC patient samples, tumor cells express CD137 through the activation of K-Ras and MAPK activation (39). In the present data set, CD137 was not associated with improved anti-tumor response. CD27, a marker involved in T memory cell development and increased cytotoxicity in lymphocytes (42), was only upregulated with the vIL-2 virus, indicating an additional advantage in the context of long term anti-tumor response. In summary, these results suggest the vIL-2 virus mediates effective co-stimulatory mechanisms to engage T-cell anti-tumor response.

In concordance with these findings, Ad5/3-E2F-d24-vIL2 treatment supported TCR signaling and T-cell degranulation that led to the best tumor response. The vIL-2 virus resulted in consistent upregulation of the CD3 co-stimulatory family (*CD3D, CD3E, CD3G*), of which CD3G is noteworthy as it actively participates in TCR expression (43). Some key downstream signaling molecules (LCK, ITK, ZAP70) (44) were similarly expressed by both armed viruses. However, *SAP* upregulation was only detected following Ad5/3-E2F-d24-vIL2 therapy. *SAP* (*SH2D1A*) belongs to a family of adapter proteins involved in the docking of T cells to their targets and T-cell cytotoxicity (45). Our results corroborate previous studies showing that SAP expression facilitates T-cell interactions and increases T-cell cytotoxicity (45, 46). In fact, our data on TILs shows prominent upregulation of perforin 1 (*PRF1*), granzymes K (*GZMK*) and M (*GZMM*), which are key for lymphocyte anti-tumor cytotoxicity (47). Surprisingly, vIL-2 seems to exert a direct effect on T-cell anchoring (*SAP*) and T-cell toxicity (*GZMM*), since a strong correlation was observed between the expressions of these genes with Ad5/3-E2F-d24-vIL2.

For other vIL-2 approaches reduction of TReg counts has been suggested as a key mechanism (9, 25, 48). In contrast, in the hamster PDAC model used here, TRegs did not appear as important suppressive player as myeloid cells. Besides classically TReg-associated genes like *CD25* and *FOXP3 (* 9, 48), cytokine genes associated with TReg activity (*IL-10*) or its repression (*IL-6*) were downregulated by both armed viruses (49). In summary, Ad5/3-E2F-d24-vIL2 virus therapy seems an appealing approach to revert myeloid-cell mediated tumor immunosuppression.

Our data demonstrate that Ad5/3-E2F-d24-vIL2 reverted immunosuppression in hamster pancreatic cancers with systematic coordination of myeloid and T-cell mediated anti-tumor responses. The mechanism of action of Ad5/3-E2F-d24-vIL2 appears more refined than its wt IL-2 armed counterpart, as evidenced by lower levels of necrosis and fewer suppressive myeloid effects at the tumor site. Instead, the vIL-2 armed virus demonstrated stronger antigen presentation and T effector activity cells through *CD3G, SAP, GZMM* and *GZMK* expression. Based on these data, Ad5/3-E2F-d24-vIL2 constitutes a promising candidate for oncolytic immunotherapy of immunosuppressive tumors.

## Data Availability Statement

The datasets presented in this study can be found in online repositories. The names of the repository/repositories and accession number(s) can be found in the article/**Supplementary Material**.

## Ethics Statement

The animal experiments and procedures here described were performed in accordance with the Experimental Animal Experimental Board (ELLA) of the University of Helsinki and the Provincial Government of Southern Finland.

## Author Contributions

DQ, SZ, JS, VC-C, RH, and AH designed the experiments. DQ, SZ, JS, VC-C, TK, and SB conducted the experiments. DQ, JS, VC-C, RH, MA, AK, and AH analyzed the results. All authors contributed to the article and approved the submitted version.

## Funding

This work was supported by the Finnish Cultural Foundation (00200899), Jane and Aatos Erkko Foundation, HUCH Research Funds (VTR), Sigrid Juselius Foundation, Finnish Cancer Organizations, University of Helsinki, TILT Biotherapeutics, Novo Nordisk Foundation, Päivikki and Sakari Sohlberg Foundation, and Finnish Society of Sciences and Letters.

## Conflict of Interest

AH is shareholder in Targovax ASA (Oslo, Norway) and in TILT Biotherapeutics (Helsinki, Finland). AH, JS, VC-C, and RH are employees in TILT Biotherapeutics (Helsinki, Finland).

The remaining authors declare that the research was conducted in the absence of any commercial or financial relationships that could be construed as a potential conflict of interest.
