# Supplementary material for: Oncolytic Adenovirus Coding for a Variant Interleukin 2 (vIL-2) Cytokine Re-Programs the Tumor Microenvironment and Confers Enhanced Tumor Control
Source: Front Immunol. 2021 May 18;12:674400. doi: 10.3389/fimmu.2021.674400 (PMC8168464; doi:10.3389/fimmu.2021.674400)
Supplement: Supplementary file 1 [file DataSheet_1.pdf]

Supplementary Material

Target Gene	Primers and Probes
Hamster IL-2 gene(16)	Forward [5´-GTGCACCCACTTCAAGCTCTAA-3´]
	Reverse [5´-AAGCTCCTGTAAGCTCAGCAGTAAC-3´]
	Probe [6-Fam-AGG AAACCCAGCAGCACCTCGAGC-BHQ-1]
Wild type human IL-2 transgene	Forward [5´-ACTTTCACTTAAGACCCAGGGA-3´]
	Reverse[5´- CACACATGAATGTTGTTTCAGATCC-3´]
	Probe [6-Fam-CGTAATAGTTCTGGAACTAAAGGGA-BHQ-2.]
Human IL-2 variant transgene	Forward [5´-CAAACCTCTGGAGGAAGTGCT-3´]
	Reverse [5´- ACGTTGATATTGCTCACCACG-3´]
	Probe [6-Fam-AACTTTCACTTCGACC-BHQ-2]
Hamster GAPDH gene(22)	Forward[5´-CACCGAGGACCAGGTTGTCT-3´]
	Reverse[5´-CATACCAGGAGATGAGCTTTACGA-3´]
	Probe[6FAM-CAAGAGTGACT CCCACTCTTCCACCTTTGA(TAMRA)]

Supplementary Table 1. RT-qPCR target genes, primers and probes sequences used for the relative mRNA expression analysis.

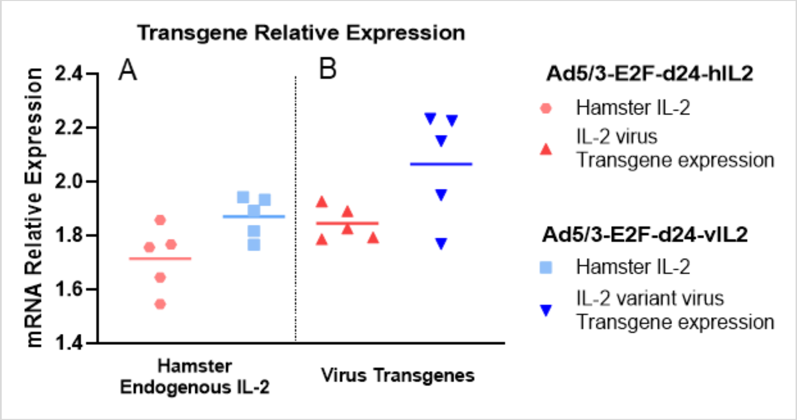

Supplementary Figure 1. IL-2 cytokine mRNA expression of in groups treated with cytokine-armed viruses relative to the housekeeping gene. (A) Endogenous hamster wt IL-2 cytokine mRNA relative expression. (B) Virus transgenes' relative mRNA expression.

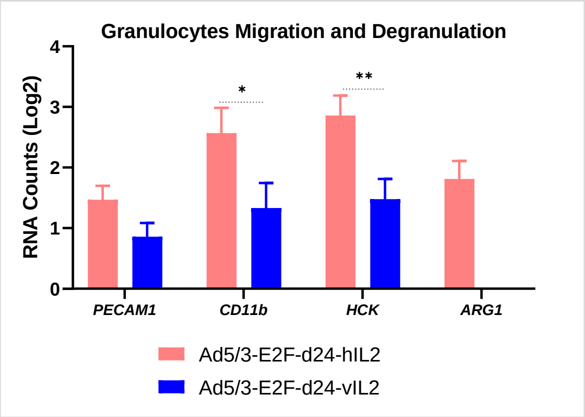

Supplementary Figure 2. Granulocytes migration and degranulation molecular gene mRNA (Log2) expression analysis in the viruses treated tumours of genes associated. Unpaired t-test with Welch´s correction. All data is presented as mean+SEM. *p<0.05 and **p<0.01.

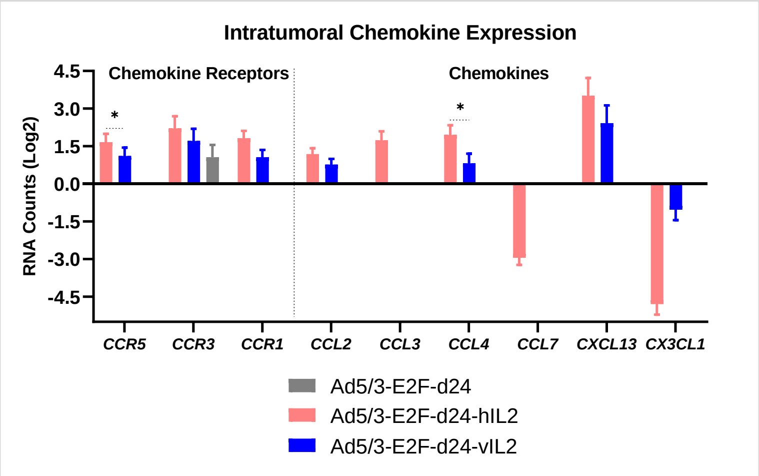

Supplementary Figure 3. Intratumoral chemokines genes' mRNA (Log2) expression analysis in the viruses treated groups. Chemokine receptors (left side) and chemokine genes (right side). All data is presented as mean+SEM. *p<0.05.

	Viruses Treatments					
	Ad5/3-E2F-d24		Ad5/3-E2F-d24-hIL2		Ad5/3-E2F-d24-vIL2	
Genes	Log2 fold change	P-value	Log2 fold change	P-value	Log2 fold change	P-value
ARG1	0.0297	0.922	1.81	1.73E-05	0.503	0.112
ATM	-0.196	0.234	-1.31	4.49E-07	-0.665	0.000723
BAX	-0.0734	0.685	-1.34	1.26E-06	-0.609	0.00349
BID	-0.036	0.921	-1.72	0.000203	-0.526	0.159
CASP3	-0.0794	0.755	-2.52	2.60E-08	-0.883	0.00279
CASP8	-0.12	0.623	-2.53	1.34E-08	-0.679	0.0117
CCL2	0.172	0.461	1.19	8.05E-05	0.768	0.00384
CCL3	-0.0577	0.874	1.74	0.000173	0.334	0.365
CCL4	0.213	0.586	1.95	0.000114	0.822	0.048
CCL5	0.0914	0.801	-0.314	0.39	0.39	0.289
CCL7	0.225	0.43	-2.95	1.29E-08	-0.163	0.566
CCL8	0.0961	0.769	0.318	0.337	0.241	0.464
CCR1	0.366	0.229	1.82	1.23E-05	1.06	0.0023
CCR3	1.06	0.0467	2.21	0.000344	1.71	0.00304
CCR5	0.255	0.459	1.66	0.000147	1.11	0.00446
CD103	-0.00602	0.989	1.24	0.0126	0.573	0.214
PD-L1	0.185	0.597	0.958	0.0129	0.489	0.173
CD27	0.243	0.545	0.105	0.793	1.03	0.0186
CD39	0.167	0.374	-0.533	0.0115	-0.114	0.546
CD3D	0.234	0.397	0.661	0.0253	1	0.00179
CD3E	0.298	0.382	0.774	0.033	1.14	0.00328
CD3G	0.229	0.505	0.653	0.0698	1	0.00865
CD40	0.522	0.213	2.39	1.98E-05	1.61	0.000974
CD45RO	0.35	0.296	1.79	4.49E-05	1.3	0.00103
CD4	0.0749	0.862	1.56	0.00191	1.37	0.0049
CD62L	0.48	0.29	1.84	0.00068	1.64	0.00177
CD7	0.238	0.515	0.854	0.0296	0.9	0.0229
CD80	0.553	0.168	2.48	7.05E-06	1.25	0.00475
CD86	0.243	0.459	2.57	5.03E-07	1.6	0.000131
CD8A	0.267	0.507	0.847	0.0467	1.22	0.0068
COX2	0.164	0.687	0.823	0.0561	-0.199	0.626
CTLA-4	0.587	0.239	2.4	0.00012	1.67	0.00299
CX3CL1	-0.108	0.8	-4.79	5.34E-09	-1.03	0.0257
CXCL10	0.385	0.383	0.662	0.143	0.814	0.0761
CXCL13	0.135	0.853	3.51	0.000149	2.42	0.0037
FCGR2B	-0.00493	0.976	0.423	0.0176	0.409	0.0209
FUT7	0.23	0.52	2.21	8.20E-06	0.961	0.0135
GZMK	0.318	0.5	0.0718	0.878	1.07	0.033
GZMM	0.219	0.589	0.494	0.232	1.05	0.0178
TIM-3	0.115	0.673	1.02	0.00153	0.728	0.0151
HCK	0.334	0.327	2.86	1.91E-07	1.48	0.000364
HMGB1	-0.0777	0.681	-1.52	3.99E-07	-0.533	0.011
IDO1	0.786	0.108	-1.62	0.00292	1.09	0.0315
IFNAR1	0.0188	0.867	0.631	3.18E-05	0.0164	0.884
IFNG	0.113	0.77	-0.231	0.551	0.336	0.388
IFNGR1	0.0576	0.609	0.577	8.04E-05	0.27	0.0263
IL10	-0.223	0.494	-2.61	4.22E-07	-1.68	7.47E-05
IL1B	0.568	0.364	3.93	7.78E-06	2.18	0.00249
IL2RG	0.393	0.185	2.11	1.31E-06	1.39	0.000155
IL6	-0.0331	0.944	-1.96	0.000673	-0.7	0.149
IRAK1	-0.0851	0.495	-0.961	8.54E-07	-0.768	1.23E-05
IRAK4	-0.0519	0.742	-1.17	1.22E-06	-0.586	0.00164
IRF1	0.056	0.793	-0.0245	0.909	-0.431	0.0567
IRF2	0.0118	0.913	-0.292	0.0146	-0.00705	0.948
IRF3	-0.0743	0.68	-1.24	3.04E-06	-0.554	0.00646
IRF4	0.608	0.281	3.11	3.07E-05	2	0.002

**IRF5**	0.127	0.637	1.69	8.02E-06	1.09	0.000758
**IRF8**	0.183	0.246	0.841	4.47E-05	0.477	0.00639
**ITGA1**	-0.0889	0.6	0.184	0.283	-0.00989	0.953
**CD11b**	0.316	0.458	2.57	1.31E-05	1.33	0.0054
**ITK**	0.252	0.4	0.651	0.0392	0.886	0.00743
**JAK1**	-0.0598	0.601	-0.918	4.17E-07	-0.513	0.000312
**JAK2**	0.0676	0.502	-0.0822	0.416	0.107	0.291
**ki67**	-0.267	0.282	-3.02	1.06E-09	-0.875	0.00217
**KLRK1**	0.358	0.364	1.17	0.0075	1.22	0.0056
**LBP**	0.0356	0.893	-2.21	2.81E-07	-0.791	0.00807
**LCK**	0.194	0.476	0.919	0.00322	1.02	0.00149
**MRC1**	0.00144	0.995	0.565	0.0189	0.429	0.0651
**MYD88**	0.0447	0.657	-0.436	0.000441	-0.12	0.243
**OAS3**	-0.174	0.739	-4.75	1.02E-07	-1.13	0.043
**PECAM1**	0.132	0.572	1.47	7.93E-06	0.857	0.00172
**PRF1**	0.303	0.311	0.5	0.103	0.625	0.046
**S1PR1**	0.0297	0.891	0.828	0.00115	0.175	0.421
**SBNO2**	-0.0627	0.744	-0.152	0.433	-0.655	0.00324
**SAP**	-0.0301	0.964	1.03	0.138	1.43	0.0458
**STAT5B**	-0.0456	0.564	-0.0293	0.71	-0.13	0.113
**SYK**	0.31	0.296	2.31	5.03E-07	1.24	0.000516
**TCF7**	-0.0102	0.954	-1.31	1.56E-06	-0.552	0.00585
**TGFB2**	-0.0219	0.847	-0.392	0.00292	-0.242	0.0455
**TGFBR2**	0.114	0.553	-0.374	0.0641	-0.357	0.076
**THBD**	0.0197	0.915	0.25	0.19	0.384	0.0512
**TICAM1**	0.0767	0.557	-0.778	1.91E-05	-0.33	0.0209
**TIGIT**	-0.0721	0.765	-0.538	0.0386	0.0735	0.761
**TLR2**	0.201	0.451	2.1	4.50E-07	0.893	0.00332
**TLR3**	0.09	0.634	-0.842	0.000386	-0.164	0.391
**TNF**	-0.0465	0.88	1.67	4.75E-05	0.247	0.428
**TNFRSF9**	0.243	0.568	3.04	1.70E-06	0.934	0.0392
**TNFSF10**	0.136	0.665	-1.19	0.00142	0.13	0.679
**TYK2**	-0.078	0.521	-0.358	0.00859	-0.325	0.0153
**VEGFA**	0.11	0.778	0.2	0.609	-0.339	0.392
**VEGFC**	-0.0411	0.867	-1.73	2.58E-06	-0.27	0.283
**ZAP70**	0.208	0.573	0.805	0.0402	0.885	0.0259

Supplementary Table 2. Panel of the genes analysed for differential expression (mRNA Log2) and their respective significance in p-values. Significantly upregulated genes are shown in green. Significantly downregulated genes are shown in red. Insignificant changes in the gene expression are shown in grey. The expression level of each gene in the treatment groups was normalized to their corresponding genes in the control (mock) group.
